# Increased Body Weight of the BAC HD Transgenic Mouse Model of Huntington’s Disease Accounts for Some but Not All of the Observed HD-like Motor Deficits 

**DOI:** 10.1371/currents.hd.0ab4f3645aff523c56ecc8ccbe41a198

**Published:** 2013-07-30

**Authors:** Andrea E. Kudwa, Liliana B. Menalled, Stephen Oakeshott, Carol Murphy, Richard Mushlin, John Fitzpatrick, Sam F. Miller, Kristi McConnell, Russell Port, Justin Torello, David Howland, Sylvie Ramboz, Dani Brunner

**Affiliations:** PsychoGenics Inc., Tarrytown, New York, USA; PsychoGenics Inc., Tarrytown, New York, USA; PsychoGenics Inc., Tarrytown, New York, USA; PsychoGenics Inc., Tarrytown, New York, USA; PsychoGenics Inc., Tarrytown, New York, USA; PsychoGenics Inc., Tarrytown, New York, USA; PsychoGenics Inc., Tarrytown, New York, USA; PsychoGenics Inc., Tarrytown, New York, USA; PsychoGenics Inc., Tarrytown, New York, USA; PsychoGenics Inc., Tarrytown, New York, USA; CHDI Foundation Inc, Princeton, New Jersey, USA; PsychoGenics Inc., Tarrytown, New York, USA; PsychoGenics Inc., Tarrytown, New York, USA; Columbia University, New York, New York, USA

## Abstract

The genome of the Bacterial Artificial Chromosome (BAC) transgenic mouse model of Huntington’s Disease (BAC HD) contains the 170 kb human HTT locus modified by the addition of exon 1 with 97 mixed CAA-CAG repeats. BAC HD mice present robust behavioral deficits in both the open field and the accelerating rotarod tests, two standard behavioral assays of motor function. BAC HD mice, however, also typically present significantly increased body weights relative to wildtype littermate controls (WT) which potentially confounds the interpretation of any motor deficits associated directly with the effects of mutant huntingtin. In order to evaluate this possible confound of body weight, we directly compared the performance of BAC HD and WT female mice under food restricted versus free feeding conditions in both the open field and rotarod tasks to test the hypothesis that some of the motor deficits observed in this HTT-transgenic mouse line results solely from increased body weight. Our results suggest that the rotarod deficit exhibited by BAC HD mice is modulated by both body weight and non-body weight factors resulting from overexpression of full length mutant Htt. When body weights of WT and BAC HD transgenic mice were normalized using restricted feeding, the deficits exhibited by BAC HD mice on the rotarod task were less marked, but were still significant. Since the rotarod deficit between WT and BAC HD mice is attenuated when body weight is normalized by food restriction, utilization of this task in BAC HD mice during pre-clinical evaluation must be powered accordingly and results carefully considered as therapeutic benefit can result from decreased overall body weight and or motoric improvement that may not be related to body mass. Furthermore, after controlling for body weight differences, the hypoactive phenotype displayed by ad libitum fed BAC HD mice in the open field assay was not observed in the BAC HD mice undergoing food restriction. These findings suggest that assessment of spontaneous locomotor activity, as measured in the open field test, may not be the appropriate behavioral endpoint to evaluate the BAC HD mouse during preclinical evaluation since it appears that the apparent hypoactive phenotype in this model is driven primarily by body weight differences.

## Introduction

Huntington’s disease (HD) is an inherited dominant autosomal neurodegenerative disorder characterized by motor deficits, cognitive decline and psychiatric manifestations 1. Typically, disease symptoms start in mid-adulthood and progress up to a terminal state within 10-20 years. In 1993, the mutation responsible for the disease was identified as an unstable expansion in the number of CAG repeats in the huntingtin gene 2.

Since the identification of this mutation, numerous mouse models of the disease have been generated 3 ^,^ 4 including a bacterial artificial chromosome transgenic mouse line (BAC HD) expressing full-length human huntingtin with a pathological polyglutamine sequence under its endogenous regulatory elements 5. More specifically, the genome of the BAC HD mouse contains the 170 kb human HTT locus modified by the addition of exon 1 with 97 mixed CAA-CAG repeats 5 ^,^ 6. The BAC HD mouse model presents robust behavioral deficits including progressive motor dysfunction with a relatively early onset as well as neuropil-associated aggregation and neurodegenerative pathology 5 ^,^ 7 ^,^ 8. Contrary to the severe wasting observed in HD patients, however, BAC HD mice show a significant increase in body weight 7 ^,^ 9.This increase in body weight may be at least partially mediated by the high level of full-length huntingtin expressed by BAC HD mice compared to their wild type (WT) controls 9. This confounding body weight phenotype is also observed in the YAC128 mouse, another full-length mutant htt model that displays motor deficits and selective neurodegenerative pathology 9 ^,^ 10 ^,^ 11 ^,^ 12 ^,^ 13 ^,^ 14 ^,^ 15 ^,^ 16, and it was demonstrated that full-length mutant htt modulates body weight in both BAC HD and YAC128 models by influencing insulin-like growth factor-1 expression 1 ^,^ 9. More specifically, increased plasma IGF-1 levels in BAC HD and YAC128 mice expressing full-length human mutant htt correlate with increased body weight and htt levels, whereas transgenic YAC mice expressing truncated mutant htt (shortstop mice) do not show elevated plasma IGF-1 and their body weight is comparable to that of WT counterparts. This increase in weight in the BAC HD mouse and other full-length htt models is therefore of potential significance when evaluating mechanisms of action and potential therapies given that body weight has been shown to be a confounding factor in the assessment of motor performance in a variety of mouse strains 17. Although the question of whether body weight affects motor function has not been directly evaluated in BAC HD mice to date, it has been suggested using different analytical approaches that the increased body weight observed in BAC HD mice does not account for the entirety of motor deficits observed in this model 5 ^,^ 7.

While the behavioral and neuropathological abnormalities observed in full-length mutant models of HD make them relevant for investigating the pathogenic mechanism of HD and to evaluate potential therapeutic strategies 7 ^,^ 18 ^,^ 19 ^,^ 20 ^,^ 21, it is clearly advantageous to better understand the extent to which body weight impacts motor performance in these transgenic mice in order to correctly design a behavioral battery for assessment of these animals. The potentially confounding effects of body weight could be particularly misleading in the preclinical realm as potential therapeutic interventions may modulate body weight in treated mice, and therefore, may elicit non HD-specific alterations in motor performance.

In order to dissociate the disease-like behavioral deficits exhibited by BAC HD mice from those effects mediated by the weight increases seen in these mice, we directly evaluated the differences in behavioral performance of BAC HD mice and wild type littermates following restricted feeding in comparison to the performance of matched free-feeding animals.

## Materials and Methods

**Subjects**

Female hemizygous (BAC HD) and wild type (WT) mice used in the current study were generated by crossing male hemizygous BAC HD mice [FVB/nJ F1 background; CHDI-80000007-(3)(1)] with female C57BL/6J mice. All experimental animals were ear notched at around 10-15 days, and tail samples were collected for genotyping purposes. Mice were then weaned and subcutaneously implanted with RFID electronic chips (DataMars, OH) around 3-4 weeks of age for identification purposes. Mice were housed in OptiMICE® cages (Animal Care Systems, CO) with wood shavings and environmental enrichment provided in the form of a plastic bone, shredded paper and a plastic play tunnel. Temperature (68-76^o^C), humidity (30-70%) and a 12:12 reverse light-dark cycle (lights off 13:00 EST) were controlled and monitored daily.

All animal care was performed in accordance with the United States Public Health Service Policy on Humane Care and Use of Laboratory Animals, and all procedures were approved by the Institutional Animal and Use Committee of Psychogenics, Inc. (PHS OLAW animal welfare assurance number A4471-01), an AAALAC International accredited institution (Unit #001213).

**Body Weight and Food Restriction**

Prior to the onset of food restriction, all animals had *ad libitum* access to food (Purina 5001) and water, and mice were weighed weekly.

Food Restriction. Starting at 36 weeks of age for cohort 1 (tested at 65/66 wks of age; n=14-17/genotype/food group) and at 58 weeks of age for cohort 2 (tested at 95/96 wks of age; n=16/genotype/food group), the animals were housed two per cage with a female BAC HD and a female WT mouse in each cage. A subset of each genotype was then placed on a restricted diet. More specifically, BAC HD and WT cage mates within each cohort were placed onto a restricted diet with the level of food restriction gradually increasing over a period of several months. Once the body weight of the BAC HD mice undergoing food restriction reached the point where it matched 85% of the WT free-feeding body weight average, the BAC HD mice were found to consume food amounts in a 30-min free feeding test comparable to intake levels displayed by WT mice. Both WT and BAC HD mice in the restricted group were then maintained at their new target weights by daily provision of controlled quantities of food (BIO-SERV 500 mg pellets). Even when intake was restricted, however, BAC HD mice remained slightly heavier than WT controls at 65wks of age, but weighed less than WT at 95 weeks of age. Following the conclusion of testing, all experimental mice were returned to *ad libitum* food access. Water was provided *ad libitum* at all times.

**Behavioral testing**

Two cohorts of female mice were used in the present study. On the day of testing, mice were transported in their home cages from the colony room to the behavioral testing room and allowed to acclimate to the experimental room for at least one hour prior the beginning of the experiment. Experimenters were blind to genotype.

**Rotarod.** Female mice were tested over 3 consecutive days during the light phase. Each daily session included a training trial of 5 min at 4 RPM on the rotarod apparatus (Rotamex, OH). One hour later, the animals were tested in 3 accelerating trials during which the speed of the rod changed from 0 to 40 RPM over 300 s. Each trial lasted 5 minutes with an inter-trial interval of at least 30 min. The latency to fall from the rod was recorded, and any mouse remaining on the rod for more than 300 s was removed and returned to the cage. Such trials resulted in a maximum latency to fall value of 300 seconds.

**Open field.** Open field testing was performed during the dark phase of the diurnal cycle under red light conditions and began 1-2 h after onset of the dark phase of the diurnal cycle. Activity chambers (Med Associates Inc, St Albans, VT; 27 x 27 x 20.3 cm) equipped with infrared (IR) beams were used. Mice were placed in the center of the chamber and their behavior was recorded for 30 min. Quantitative analysis was performed on the following dependent measures: total locomotion and rearing rate in the center of the open field.

**Statistical analysis.** An alpha level of 0.05 was selected for all inferential statistics. ANOVA analysis was carried out with SAS (SAS Institute Inc.) using Mixed Effect Models. This approach was based on likelihood estimation which was more robust to missing values than moment estimation. The models were fitted separately for each age using the procedure PROC MIXED 22. For this study, Genotype, Food condition and their interactions were considered as factors in all the models. Significant Genotype x Food condition interactions evaluated were followed up with simple main effects to further identify significant genotype and food condition effects at each testing age.

In addition, planned comparisons were used to directly compare food restricted BAC HD mice against freely fed WT controls using the Pdiff option for the LS means statement in SAS Proc Mixed.

## Results

**Body Weight**

As expected, at both 65 and 95 weeks of age, BAC HD female mice weighed significantly more than did female mice, and food-deprived mice weighed less than freely fed mice in both genotype groups. Food-deprived BAC HD mice still showed a slightly, but significantly, higher body weight as compared to the WT controls on teh restricted diet (65wks: Genotype x Food condition interaction, F_(1,65)_=43.88, *p<*0.0001; Genotype main effect, F_(1,65)_=269.03, *p<*0.0001; Food condition main effect, F_(1,65)_=654.98, *p<*0.0001; Simple main effects, *ps<*0.05, Figure 1A; 95wks: Genotype x Food condition interaction, F_(1,60)_=174.79, *p<*0.0001; Genotype main effect, F_(1,60)_=463.91, *p<*0.0001; Food condition main effect, F_(1,60)_=654.98, *p<*0.0001; Simple main effects, *ps<*0.05; Figure 1B).

**Body weight of female BAC HD and WT mice following either food restriction (FR) or free feeding at 65 (Panel A) and 95 (Panel B) weeks of age:**
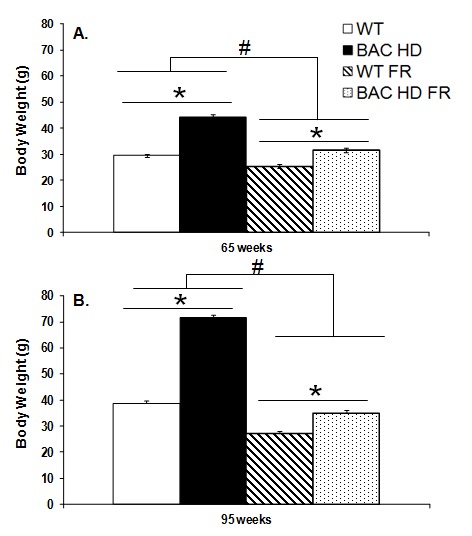
Data are presented as mean ± S.E.M. * significant effect of genotype; # significant effect of food condition, p<0.05.

**Rotarod**

At both 66 and 95 weeks of age, female BAC HD mice remained on the accelerating rotarod for a shorter period of time than did female WT mice, regardless of food condition (66wks: Food condition main effect, F_(1,65)_=82.60, *p<*0.0001, Figure 2A; 96wks: Genotype main effect, F_(1,60)_=205.33, *p<*0.0001, Figure 2B). Mice undergoing food restriction also remained on the rotarod longer than free feeding animals, regardless of genotype (66 wks: Food condition main effect, F_(1,65)_=8.81, *p<*0.01; Genotype x Food condition interactions, NS; Figure 2A; 96 wks: Food condition main effect, F_(1,60)_=57.87, *p<*0.0001; Genotype x Food condition interactions, NS; Figure 2B).

**Performance of female BAC HD and WT mice in the rotarod task following either food restriction (FR) or free feeding at 66 (Panel A) and 96 (Panel B) weeks of age.**
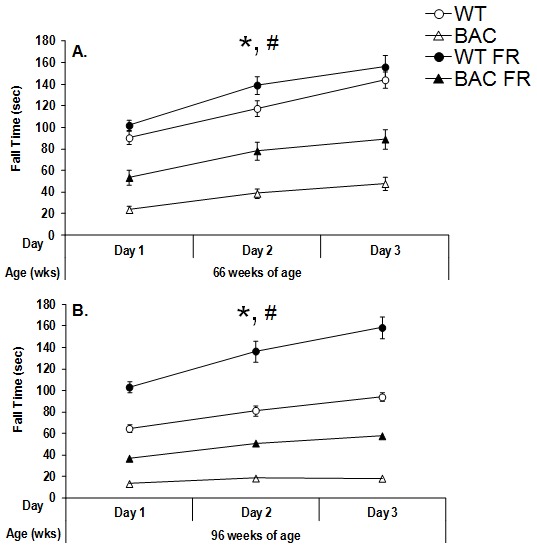
Data are presented as mean ± S.E.M. * significant effect of genotype; # significant effect of food condition, p<0.05.

Furthermore, planned comparisons comparing the performance of food restricted BAC HD mice relative to free feeding WT animals showed that food restricted BAC HD mice fell from the rotarod significantly faster than did the freely fed WT group, despite the fact that BAC HD mice undergoing food restriction are actually lighter than freely fed WT mice at the older age (*p<*0.0001). More specifically, under free feeding conditions, the fall time of the BAC HD mice was about 70% and 80% less than that exhibited by WT mice at 65 and 96 weeks of age, respectively. Under food restricted conditions, even though the relative differences in fall times between the genotypes were attenuated, the BAC HD mice still exhibited fall times that were 45% and 65% less than those displayed by WT mice at 65 and 96 weeks of age, respectively. Therefore, even though the degree of the genotype difference is larger and more powerful under free feeding conditions, these data demonstrate that robust genotype effect between BAC HD and WT mice under food restricted conditions that is not directly attributable to differences in body weight.

**Open Field - Total Distance Traveled**

At 66 weeks of age female BAC HD mice were hypoactive in comparison to WT counterparts, but only under the free feeding food condition (Genotype x Food condition interaction, F_(1,57)_=5.13, *p<*0.05; Food condition main effect, F_(1,57)_=25.96,* p<*0.0001; Simple main effects, ps<0.05; Figure 3A). At 95 weeks of age, BAC HD mice were hypoactive compared to WT counterparts in both the free feeding and restricted feeding conditions, although the genotype difference was attenuated in the food restricted group (Genotype main effect, F_(1,60)_=28.70, *p<*0.0001; Food condition main effect, F_(1,60)_=129.34, *p<*0.0001; Genotype x Food condition interaction, F_(1,60)_=3.86, *p<*0.05; Simple main effects, *ps<*0.05; Figure 3B).

Planned comparisons of total locomotor activity at both ages, however, revealed that food restricted BAC HD mice actually ambulated a greater total distance, and thus were hyperactive in the open field at both ages compared to WT mice receiving food *ad libitum* despite still being slightly heavier at the younger age (*ps<*0.05).

**Total locomotor activity (Panels A, B) and rearing rate in the center of the open field (Panels C, D) of female BAC HD and WT mice in the open field test under either food restricted (FR) or free feeding conditions at 66 (Panels A, C) and 95 (Panel B, D) weeks of age.**
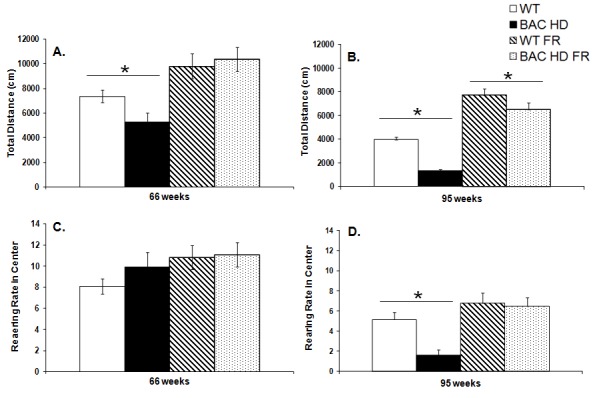
Data are presented as mean ± S.E.M. * significant effect of genotype; # significant effect of food condition, p<0.05.

**Open Field - Rearing Rate in Center of the Open Field**

At 66 weeks of age, female BAC HD and WT mice showed similar levels of rearing behavior in the center of the open field within both diet conditions (Figure 3C). In contrast, at 95 weeks of age, BAC HD mice in the free feeding condition exhibited less rearing behavior than WT mice. No genotype difference was observed in the groups undergoing food restriction (Genotype main effect, F_(1,60)_=5.49, *p<*0.05; Food condition main effect, F_(1,60)_=16.02, *p<*0.001; Genotype x Food condition interaction, F_(1,60)_=3.99, *p=*0.0504; Simple main effects, *ps<*0.05, Figure 3D).

Planned comparison of rearing rates in the center of the open field at 66 and 95 weeks of age revealed that food restricted BAC mice and freely fed WT mice did not differ in rearing frequency at either age (*p=*0.0729 and 0.2450 for 66 and 95 weeks, respectively), thus further demonstrating that the decreased rearing behavior observed in the center of the open field under free feeding conditions is attributable to body weight.

## Discussion

Genetic manipulations employed when developing animal models of neurodegenerative disease can elicit non-specific effects that result in phenotypic differences that are not attributable to the disease itself. These non disease-specific effects, if not known or controlled for, may then confound the ability to evaluate potential treatments and therapies. We demonstrate here that the rotarod performance displayed by BAC HD mice is indeed modulated by body weight, but also show that the behavioral deficit is attributable to the huntingtin mutation. The motor deficit revealed by the rotarod task remains robust over and above body weight, and the genotype differences between BAC HD and WT mice remained significant after food restriction. Furthermore, food restricted BAC HD mice at 65 weeks of age still performed worse on the rotarod task than did than free feeding WT mice. Therefore, the motor deficits observed in BAC HD mice in the current study using the rotarod task seem directly related to the HD mutation. Furthermore, the observed genotype difference replicates previous analyses in the BAC HD FVB/n line (as opposed to the FVB/nJ F1 background used here) in which the performance of a subset of male BAC HD FVB/n animals with body weights comparable to WT control males was similar to the performance of heavier BAC HD mice while still remaining significantly worse than male WT counterparts 5 ^,^ 7. It must be noted for the purposes of preclinical therapy screening, however, that the degree of difference between the performance of BAC HD and WT mice was attenuated under restricted dietary conditions which will have to be taken into account when powering future studies.

In contrast, the locomotor deficits typically observed in the open field test in the BAC HD animals do appear to be a result of increased body weight, as the phenotype was rescued simply by food restriction. In terms of the locomotor activity, where no differences were detected between the BAC HD and WT controls in the food regulated group, it is possible that the lack of a genotype difference could be related to a ceiling effect, at least in the 66 week old group, since the overall activity levels were rather high. However, the mice in the 95 week test cohort were significantly less active than the young cohort, even under food restriction, suggesting that lack of activity differences between the BAC HD and WT food restricted mice does indeed reflect the absence of a locomotor phenotype in these mice.

It has been shown that dietary restriction extends lifespan and attenuates both motor and cognitive performance in rodents 23 ^,^ 24 ^,^ 25. More specifically, food-restriction of aged mice eliminates locomotor differences from young control animals, with no apparent impact on other age-related declines in grip strength or balance beam performance 23. In the current study, female mice were enrolled as body weight differences between BAC HD and WT mice on the FVB/n background under free feeding conditions were previously shown within gender to be greater in females than in males 7. The current results show a clear overall effect of food restriction on the activity displayed by all female mice in both the rotarod and open field assays, with food restricted mice generally presenting higher levels of locomotor activity than free-feeding counterparts as is consistent with published reports. While the current study only assessed female BAC HD animals, food restriction performed in the HD-N171-82Q mouse model of HD, which express human N-terminal truncated huntingtin with 82 polyglutamine repeats driven by the mouse prion promoter 26, also resulted in a delay in the onset of motor deficits as well as a normalization of glucose metabolism and brain BDNF levels 27. It is conceivable that the mechanisms underlying the diet-specific energizing effects on locomotion resulted in an enhanced motor deficit in the BAC HD mice in the current study, as compared to WT mice, thus resulting in a disappearance of the hypoactive locomotor phenotype under restricted intake conditions (a two-hit hypothesis). It is more parsimonious, however, to assume that the hypoactive locomotor phenotype and its disappearance were simply related to body weight (a one-hit hypothesis).

Overall, our findings confirm that the behavioral deficits observed in the female BAC HD mice using the the rotarod task are in part attributable to the huntingtin mutation, and therefore reflect an HD-specific motor deficit. In contrast, these data discourage the inclusion of the open field test in preclinical testing employing the BAC HD mouse, since it appears that the apparent hypoactive phenotype is driven by body weight differences rather than by a disease-like process. In addition, our results suggest that reduction in body weight, at least through food regulation, may lead to significant reductions in phenotype effect sizes even in the rotarod. This attenuation of the genotype difference is therefore of concern in the context of assessing the therapeutic potential of a compound, not only because there is a reduced window for evaluating potential compound effects on behavior, but also because a common undesirable side effect of many drug candidates is modulation of body weight.
